# High inter-observer reliability in standardized ultrasound measurements of subcutaneous adipose tissue in children aged three to six years

**DOI:** 10.1186/s12887-020-02044-6

**Published:** 2020-04-02

**Authors:** A. Kelso, W. Müller, A. Fürhapter-Rieger, M. Sengeis, H. Ahammer, J. M. Steinacker

**Affiliations:** 1 Division of Sports and Rehabilitation Medicine, Ulm University Medical Center, Frauensteige 6, Haus 58/33, 89075 Ulm, Germany; 2 Institute of Biophysics, Medical University of Graz, Neue Stiftingtalstraße 6, 8010 Graz, Graz, Austria

**Keywords:** Body composition, Body fat, Ultrasound imaging, Preschool children, Precision

## Abstract

**Background:**

A procedure to measure subcutaneous adipose (SAT) using brightness-mode ultrasound has recently been standardized and applied to various groups of adults including underweight, overweight and obese adults. High reliability of this procedure was found in each of the examined groups. The purpose of this study was to determine inter-observer reliability of the standardized brightness-mode ultrasound measurement of uncompressed SAT in three to six-year-old children.

**Methods:**

Three experienced observers independently captured the ultrasound images at the eight standardized measurement sites in each of the 20 children and evaluated their images using an interactive software that detects the SAT contour and automatically measures multiple thicknesses in each image; the mean of these represents SAT thickness at a given site. The children were aged 4.9 ± 1.0 years; their body mass index ranged from 13.6–17.7 kgm^− 2^. Sound speed was set to 1450 ms^− 1^ for SAT.

**Results:**

SAT thickness sums with fibrous structures included (*D*_I_) ranged from 25.7–86.4 mm, mean *D*_I_ was 48.1 ± 15.5 mm. For *D*_I_, resulting from 160 measurements by each observer, the intra-class correlation coefficient was 0.998 (95% confidence interval 0.980–0.999), standard error of the estimate was 1.1 mm, and 95% limits of agreement were within ±2.1 mm. The median difference in *D*_I_ was 0.8 mm, i.e. about 1.9% of mean *D*_I_.

**Conclusions:**

Inter-observer results in children are comparable to previously described high reliability in adults. This method, which provides a technical thickness measurement accuracy of about 0.1 to 0.2 mm, enables monitoring of subcutaneous adipose tissue in children with a similarly high reliability as was obtained in adults previously.

**Trial registration:**

German Institute of Medical Documentation and Information, German Clinical Trials Register (DRKS) ID: DRKS00010089; Date 24/02/2016.

## Background

Body composition and growth are important determinants of childhood health [1]. Although childhood overweight and obesity is associated with serious health problems and the risk of premature illness and death later in life, prevalence rates continue to increase [2, 3]. The United Nations International Children’s Emergency Fund, the World Health Organization (WHO) and the World Bank Group recently published updated estimates on the nutrition status in children under five years of age [3]: in 2017, 38 million children worldwide were estimated to be overweight; at the same time, 7.5% of children around the globe were effected by wasting (i.e., approximately 50 million children were too thin for their height) [3].

These estimates were derived from measurements of body mass and height and compared to normative growth standards [1–3].

In addition to the analysis of body height (*h*) and body mass (*m*), there are several other anthropometric measures in use for determining relative body weight and body composition in adults and in children [1, 4–6]. Many epidemiological studies focus on indices such as the body mass index (BMI) (*m*/*h*^2^) [2, 7–9], which is a measure of relative body weight, but not a useful tool for determining the individual’s body composition because it cannot distinguish between body fat and muscle mass [1, 4]. A similar BMI in different individuals may not correspond to a similar amount of body fat. Furthermore, as stated by the WHO Expert Committee: ‘Problems in using the BMI further arise in individuals whose shape differs from the norm, particularly in individuals whose legs are shorter or longer than might be expected for their height’ [5]. As an alternative measure for relative body weight, the mass index MI = 0.53 *m*/(*h*·s) has been proposed, which considers the individual’s sitting height (*s*) and thus, implicitly, the leg length [10–12]. Nonetheless, both BMI and MI measure relative body weight and are not useful for determining body fat content [4, 13].

A widely used approach for assessing body fat, specifically subcutaneous adipose tissue (SAT), is the measurement of skinfolds. Skinfold thickness is composed of a double fold of compressed adipose tissue and skin [14]. Skinfold measurement is a low-cost method of regional body fat assessment, but has inherent methodological shortcomings. Errors in the collection of raw skinfold data are expected due to site-specific compression of adipose tissue and individual variations in the elasticity of the skin [15, 16]. Additionally, researchers and practitioners should be wary of prediction equations that estimate total body fat percentage from skinfolds on the individual level [1, 14]. The accuracy and validity of these equations relies on several assumptions: skinfolds are of constant compressibility, skin thickness is the same at all sites, fat fraction and patterning of SAT are constant, as is the ratio of external to internal adiposity. As stated by Marfell-Jones et al. and by Clarys et al.: ‘none of these assumptions hold true’ [14, 15]. These shortcomings explain the large discrepancies between skinfold and ultrasound (US) measurements [17].

A new approach has recently been introduced which results in highly accurateand reliable measurements of uncompressed SAT in adults [15, 18]. This approach captures the skin, SAT, muscle fascia and the underlying muscle tissue using a standardized ultrasound imaging and image evaluation procedure at eight clearly defined body sites [19, 20]. When the appropriate speed of sound for the given tissue is used to determine the distance between borders, the measurement accuracy for determining tissue borders is approximately 0.1–0.2 mm at 12–18 MHz probe frequency [12, 15, 19]. The reliability of this technique has been tested previously in various study populations [12, 18–20]. Determining inter-observer reliability in lean individuals and physically well-trained athletes with sums of SAT thicknesses including embedded fibrous structures (*D*_I_) ranging from *D*_I_ = 10 to 50 mm, 95% of the values among experienced observers were found to be within ±1.0 mm from the mean [19]. In a group of lean to obese adults with *D*_I_ ranging from 12 to 245 mm, 95% of repeated observer measurements were within ±2.2 mm from the mean [20]. In a subgroup with *D*_I_ ranging from 12 to 77 mm, 95% of values were within ±1.4 mm from the mean, and in a second subgroup with *D*_I_ ranging from 53 to 245 mm, 95% of values were within ±2.9 mm from the mean [20].

In a sample of 274 preschool children, mean SAT thickness significantly differed between boys and girls, while anthropometric characteristics such as body mass, body height, BMI, and waist circumference did not show any significant differences [21]. Additionally, when a subset of 16 children was measured twice by one observer and *D*_I_ was compared, the intra-class correlation coefficient (ICC = 0.994) and its 95% confidence interval (95% CI: 0.983–0.998) indicated excellent intra-observer reliability [21]. Thickness sums *D*_I_ ranged from 34.8 to 112.3 mm, 95% of measurement differences in *D*_I_ were within 0.4 to 2.0 mm [21].

The standardized ultrasound technique for measuring SAT has repeatedly shown high intra-and inter-observer reliability in various groups of adults [18–20], and high **intra**-observer reliability in children [21].

However, **inter**-observer reliability studies in children are missing. The aim of this study was to bridge this gap and to compare the results found in preschool children aged three to six years to the published results in adult groups. The analysis of the inter-observer reliability will allow a large-scale implementation of this technique.

## Methods

### Participants and observers

**Fig. 1:**
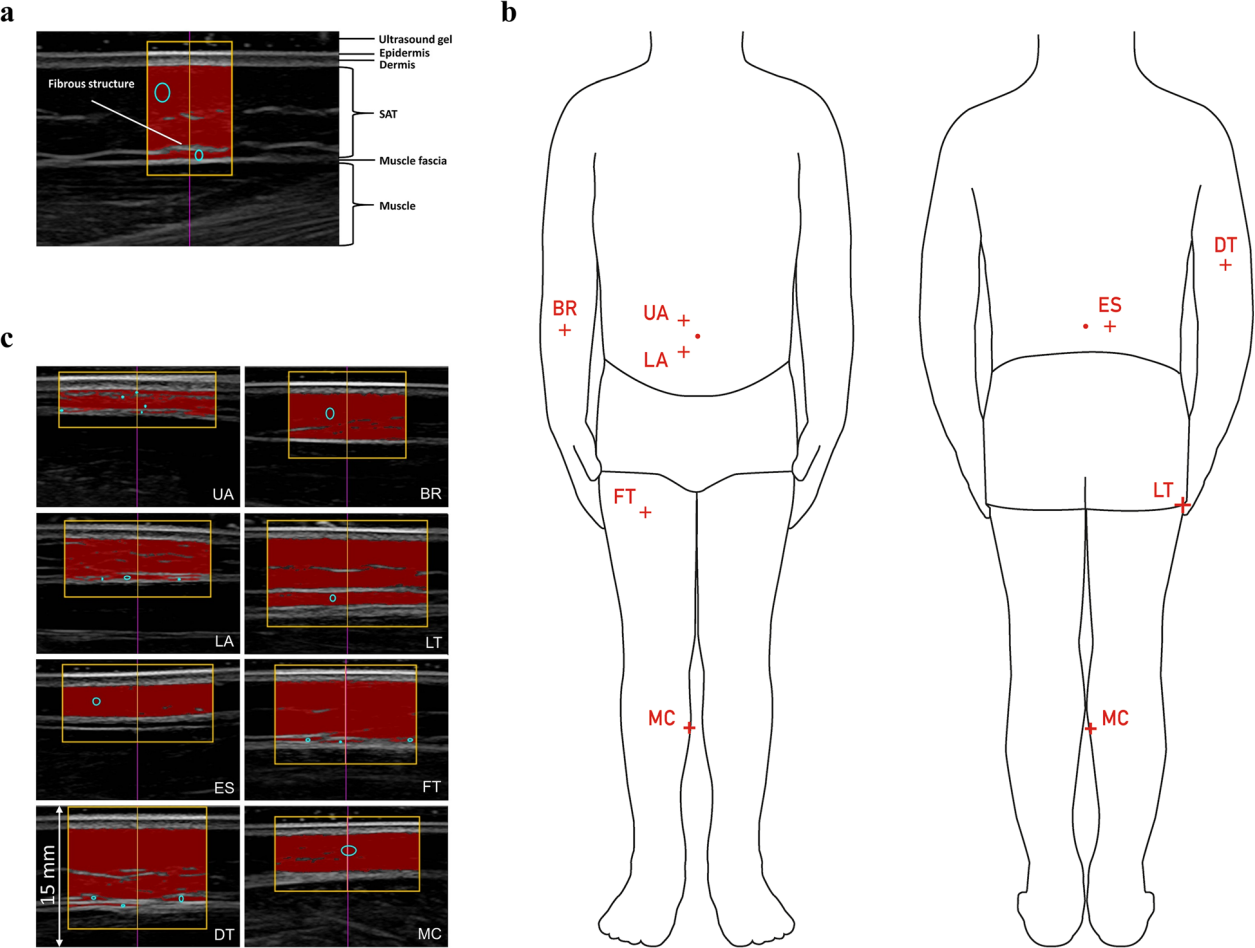
Brightness-mode ultrasound imaging of SAT. **a** Example of an ultrasound image taken at the site distal triceps. Within the region of interest, the software evaluation algorithm automatically measured subcutaneous adipose tissue thickness (SAT) along 118 vertical lines from the lower border of the dermis to the upper border of the muscle fascia. Mean SAT thickness (*d*) including embedded fibrous structures (*d*_I_) was 8.1 mm, excluding embedded structures (*d*_E_), SAT thickness was 7.5 mm. **b** Ultrasound measurement sites: upper abdomen (UA), lower abdomen (LA), erector spinae (ES), distal triceps (DT), brachioradialis (BR), lateral thigh (LT), front thigh (FT), medial calf (MC). **c** Series of evaluated ultrasound images: image depth was 30 mm, sum of SAT thicknesses at the eight sites including embedded structures (*D*_I_) was 36.1 mm (*D*_E_ = 33.0 mm). Thicknesses of individual sites *d*_I_ (*d*_E_ in parentheses) at UA: 2.1 mm (1.6 mm), LA: 3.8 mm (3.4 mm), ES: 2.9 mm (2.9 mm), DT: 7.2 mm (6.4 mm), BR: 4.4 mm (4.2 mm), LT: 6.3 mm (5.5 mm), FT: 5.9 mm (5.5 mm), MC 3.5 mm (3.5 mm)

In the *Health Survey*, an evaluation study of the preschool-based health promotion program *Join the Healthy Boat* in Southwest Germany, ultrasound measurements of SAT were performed as part of body composition analysis [21, 22]. The inter-observer reliability analysis presented here took place within the framework of the evaluation study, for which an additional preschool was recruited. The *Health Survey* was registered at the German Clinical Trials Register (DRKS) operated by the German Institute of Medical Documentation and Information, Cologne, Germany (ID: DRKS00010089) and approved by the ethics committee of Ulm University (application number 188/15) and is in accordance with the Declaration of Helsinki. Written consent to participate in the reliability analysis was given by the parents of 20 children (40% boys) aged 4.9 ± 1.0 years. Three observers (AF, AK, MS) certified by the International Association of Sciences in Medicine and Sports (www.iasms.org) performed the ultrasound measurements of SAT (Fig. 1a). The three observers had previously measured over 300 individuals using the standardized ultrasound approach [19]. The sites were marked on the right side of the body (Fig. 1b) by one of the observers and double-checked by one of the other two observers. Each of the three observers captured the eight ultrasound images of each of the 20 children and evaluated these 160 images, without having access to the results of the other two observers. The example of one such measurement series is shown in Fig. 1c.

### Anthropometry

Anthropometric measurements were performed in accordance with the International Standards for Anthropometric Measurements [23]. Body height (*h*), sitting height (*s*) were measured to the nearest 0.1 cm, and body mass (*m*) to the nearest 0.05 kg. The BMI (*m/h*^*2*^) and the MI (0.53 *m/(h·s)*) [4, 11, 12] were calculated. The MI considers individual sitting height *s* for assessing relative body weight. In individuals with long legs, the MI is higher than the BMI and vice versa. For a person with a Cormic index *C = s/h = 0.53*, representing mean leg length, the BMI and the MI are equal [4, 11, 12].

### Site marking

The observers marked the eight standard sites on the right side of the participants’ body. These sites are defined with respect to the individual’s body height (*h*) [19]. In this group of children, the same percentages defined in adults were used without modifications. Fig. 1b shows the eight standard sites. The upper abdomen, lower abdomen, and lateral thigh were marked with the participant standing; the erector spinae was marked in an upright sitting position; distal triceps, brachioradialis were marked with the forearm supported by a table and the upper arm positioned vertically; front thigh and medial calf were marked with the foot supported such that the upper leg was positioned horizontally. The detailed description and illustration of the site marking has been published previously [19].

### Ultrasound

Three observers captured the brightness-mode ultrasound images at the eight marked measurement sites in each child independently and evaluated their 160 images. The standardized brightness-mode ultrasound imaging was performed with the participants lying in a supine, prone or rotated position [19]. The operator positioned the centre of the linear probe over the marked site and held it perpendicularly to the skin and longitudinally in the direction of the underlying muscle.

A thick layer of ultrasound gel, typically 5 mm, was used between the probe and the skin to avoid compression. The gel layer appeared as a black band on top of the ultrasound image, and underneath, the epidermis, dermis, SAT, muscle fascia, and muscle were clearly visible (Fig. 1a and c). The ultrasound systems used by the observers (CX50 Philips Ultrasound, Bothell, WA, USA; GE Logiq-e General Electric, GE Healthcare, IL, USA) with linear probes operated at 12 to 18 MHz had similar image resolution of 0.1 to 0.2 mm. The accuracy obtainable with brightness-mode (B-mode) ultrasound depends on the probe frequency, on the appropriate setting of the ultrasound system, and on the skills of the observer. Linear probes were used for quantitative measurements. Tissue compression was avoided by including a thick layer of gel between the probe and the skin [12, 15, 19]. The resolution of ultrasound imaging is determined by the ultrasound wavelength (λ): at 18 MHz probe frequency (f), a resolution of about 0.1 mm can be obtained, which is approximately equal to the wavelength. A detailed discussions of the ultrasound thickness measurement accuracy can be found in preceding publications [12, 19, 20].

Each of the three observers captured the eight images at the standardized sites in 20 children, resulting in three measurement series and a number of 3·8·20 = 480 ultrasound images, which formed the basis for this inter-observer reliability study.

### Image evaluation

The images were imported into the SAT image evaluation software (NISOS-FAT v 3.2, Rotosport, Stattegg, Austria; www.rotosport.at) to evaluate SAT thicknesses at the eight standard sites. The observers evaluated their sets of images independently. The SAT contour was detected interactively, and multiple thicknesses of SAT, typically 100 per image, were measured automatically. The robust mean of these thicknesses determines the SAT thickness at the given site. Speed of sound was set to *c* = 1450 ms^− 1^ for distance determination in SAT [24]. The semi-automatic tissue segmentation of the software was controlled visually. The software reported thickness values at each individual site (*d*) including and excluding embedded fibrous structures (indices I and E, respectively), and also calculated the sums of the eight individual sites *D*_I_ and *D*_E_, respectively. The sum of embedded fibrous structures was also calculated as *D*_I_ - *D*_E_.

### Statistical analysis

SPSS 21 (IBM Corp, Armonk, NY, USA) was used. Values were reported as mean ± standard deviation (SD). Normal distribution was tested using the Shapiro-Wilk test. The ICC and its’ 95% CI were calculated based on a two-way random effects model with average measures [25]. A linear regression analysis was performed to calculate the standard error of the estimate when comparing the individual measurement results of the three observers to their mean values; additionally, Pearson’s product-moment correlation coefficient *r* was determined. The level of significance was set to *p* ≤ 0.05. Modified Bland-Altman plots were constructed to display the individual observer differences (Δ) from their mean (*D*_MEAN_), and 95% limits of agreement were calculated as mean difference ± 1.96·SD of the differences [26]. Similar types of data agreement between multiple observers have previously been used by Jones et al. [27], and in a series of reliability studies of the ultrasound method in various groups of adults [12, 15, 18–20]. ANOVA including Levene statistics for variance homogeneity and Tukey-HSD post hoc tests was carried out to test inter-observer homogeneity.

## Results

In this group of children, mean SAT thickness sums including embedded fibrous structures (*D*_I,MEAN_), calculated from three measurement series per child, ranged from 25.7 to 86.4 mm (Table 1); the group mean value was 48.1 ± 15.5 mm. The thickness sums excluding embedded structures, *D*_E,MEAN_, ranged from 21.4 to 80.5 mm, with a group mean of 43.4 ± 15.0 mm (Table 1). Table 2 shows the *D*_I_-values of each observer and for each participant. In addition, each observer’s individual difference, Δ_I_, from *D*_I,MEAN_ is given. The respective values for *D*_E_ can be found in Additional file 1.

**Table 1:** Characteristics of the study sample (*n* = 20)

	MEAN ± SD	(range)	Unit
Age	4.86 ± 0.96	(3.10–6.36)	[year]
Height, *h*	1.09 ± 0.1	(1.0–1.3)	[m]
Sitting height, *s*	0.61 ± 0.04	(0.57–0.71)	[m]
Mass, *m*	18.5 ± 3.9	(13.5–29.1)	[kg]
Body mass index, *BMI*	15.4 ± 1.2	(13.6–17.7)	[kgm^− 2^]
Mass index, *MI*	14.5 ± 1.2	(12.5–17.1)	[kgm^− 2^]
Cormic index, *C*	56.3 ± 1.2	(53.7–59.2)	[1]
*D*_I_ OBS 1	47.0 ± 15.2	(24.9–84.6)	[mm]
*D*_I_ OBS 2	48.0 ± 15.3	(25.3–85.8)	[mm]
*D*_I_ OBS 3	49.2 ± 15.8	(26.9–88.9)	[mm]
D_I,MEAN_	48.1 ± 15.5	(25.7–86.4)	[mm]
*D*_E_ OBS 1	42.9 ± 15.1	(21.0–79.7)	[mm]
*D*_E_ OBS 2	43.8 ± 15.4	(21.3–81.1)	[mm]
*D*_E_ OBS 3	43.7 ± 14.6	(21.7–80.7)	[mm]
*D*_E,MEAN_	43.4 ± 15.0	(21.4–80.5)	[mm]

*D*_I_ sum of subcutaneous adipose tissue of the eight measured sites including embedded fibrous structures, *D*_E_ sum of subcutaneous adipose tissue excluding embedded fibrous structures, *OBS* observer, *SD* standard deviation

**Table 2:** SAT thickness sums of each participant measured by the three observers

		*D*_I_ [mm]			Δ_I_ [mm]		
*P*	*D*_I,MEAN_	OBS1	OBS2	OBS3	OBS1	OBS2	OBS3
1	25.7	24.9	25.3	26.9	−0.8	− 0.4	1.2
2	32.4	31.6	32.1	33.4	−0.8	− 0.3	1.0
3	32.4	30.9	33.2	33.0	−1.5	0.8	0.6
4	36.7	36.2	36.5	37.3	−0.5	−0.2	0.6
5	37.1	36.2	36.4	38.7	−0.9	− 0.7	1.6
6	38.0	37.1	38.8	38.0	−0.9	0.8	0.0
7	39.2	38.2	39.7	39.8	−1.0	0.5	0.6
8	39.9	38.0	40.9	40.8	−1.9	1.0	0.9
9	40.2	39.8	40.1	40.6	−0.4	−0.1	0.4
10	43.5	42.8	42.9	44.8	−0.7	−0.6	1.3
11	44.8	43.1	45.3	45.9	−1.7	0.5	1.1
12	48.7	47.9	48.4	50.0	−0.8	−0.3	1.3
13	52.6	52.3	51.9	53.6	−0.3	−0.7	1.0
14	52.7	50.5	53.0	54.5	−2.2	0.3	1.8
15	52.9	52.7	52.4	53.6	−0.2	−0.5	0.7
16	53.4	53.2	52.0	55.0	−0.2	−1.4	1.6
17	61.2	59.7	61.5	62.4	−1.5	0.3	1.2
18	64.9	63.7	65.1	65.9	−1.2	0.2	1.0
19	78.5	76.5	78.4	80.7	−2.0	−0.1	2.2
20	86.4	84.6	85.8	88.9	−1.8	−0.6	2.5

Individual thickness sums of subcutaneous adipose tissue (SAT) including embedded fibrous structures (*D*_I_) for each of the 20 participants (P) and for each of the three observers (OBS), and and the means of the three measurements (*D*_I,MEAN_). Individual observer differences from the mean were calculated as: Δ_I_ = *D*_I_ - *D*_I,MEAN_

**Fig. 2:**
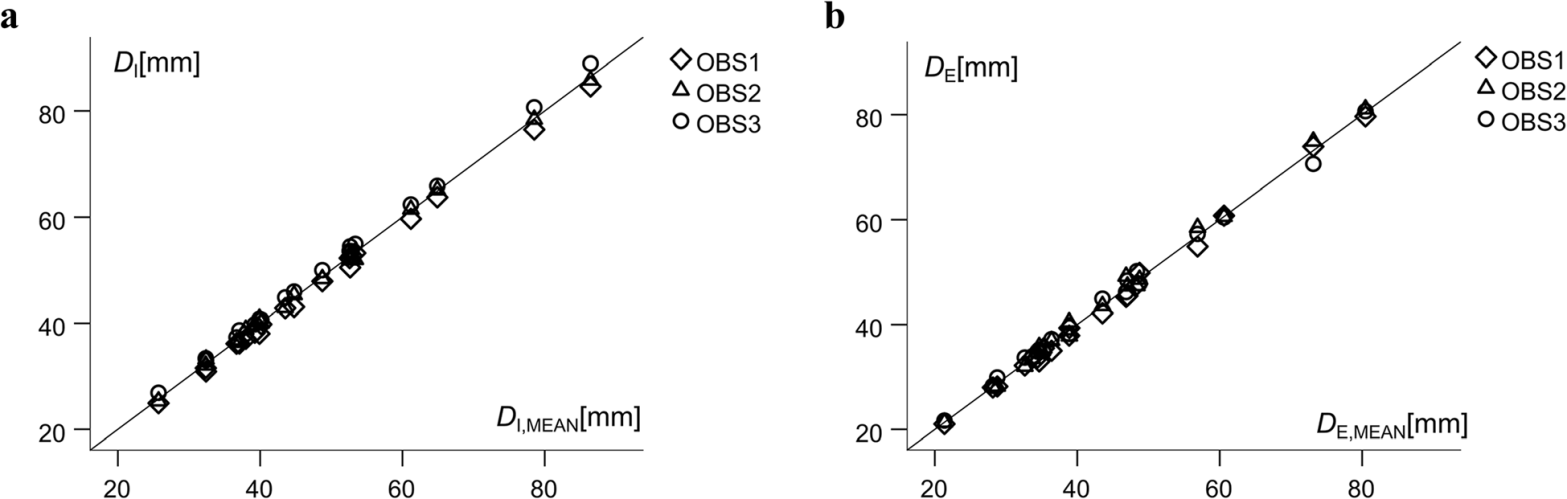
SAT thickness sums from eight sites measured three times in each of the 20 participants. **a** SAT thickness sums including embedded fibrous structures (*D*_I_) plotted against the mean value of the three observers. Pearson’s correlation coefficient *r* = 0.997, standard error of the estimate (SEE) = 1.1 mm, and intra-class correlation coefficient (ICC) = 0.998 (95% CI: 0.980–0.999). **b** SAT thickness sums excluding embedded fibrous structures (*D*_E_). *r* = 0.998; SEE = 1.02; ICC = 0.998 (95% CI = 0.995–0.999)

Figure 2a shows the *D*_I_-values measured by the three observers plotted against *D*_I,MEAN_ for each of the 20 participants. The ICC was 0.998 (95% CI: 0.980–0.999), the standard error of the estimate was 1.1 mm, and Pearson’s *r* was 0.997. The inter-observer results for *D*_E_ were: ICC = 0.998 (95% CI: 0.995–0.999), standard error of the estimate = 1.0 mm, and Pearson’s *r* = 0.998 (Fig. 2b).

**Fig. 3:**
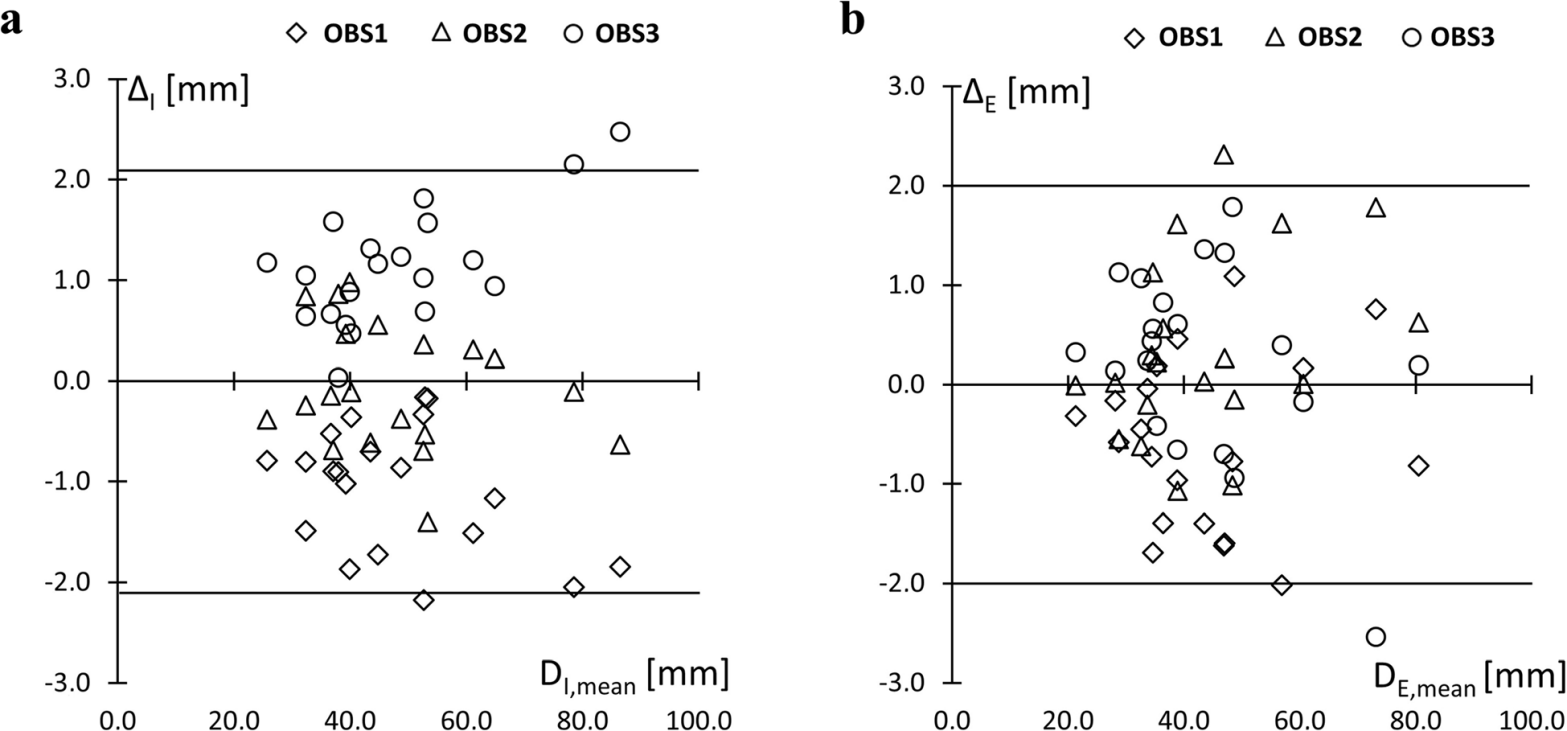
Observer differences from the mean. Individual observer differences (Δ) from the mean of the three measurements (*D*_MEAN_) are shown for each participant (see Table 2). **a** Individual observer differences from *D*_MEAN_ including embedded structures (*D*_I,MEAN_) calculated as Δ_I_ = *D*_I_–*D*_I,MEAN_ are shown. Standard deviation (SD) of observer differences was 1.1 mm, 95% of the measurements were between ±2.1 mm (limits of agreement). **b** Individual observer differences Δ_E_ = *D*_I_– *D*_E,MEAN_ are shown. SD of observer differences was 1.0 mm, 95% of measurements were between ±2.0 mm (limits of agreement)

The individual observer differences Δ_I_ from *D*_I,MEAN_ are plotted in Fig. 3a. The SD of observer differences from *D*_I,MEAN_ was 1.1 mm, 95% limits of agreement were ± 2.1 mm (1.96·SD). Accordingly for *D*_E_: SD = 1.0 mm, 95% limits of agreement were ± 2.0 mm (Fig. 3b). Variance homogeneity (Levene test) was given with *p* = 0.985. ANOVA yielded no differences between observers with *p* = 0.904 and post hoc tests (Tukey-HSD) *p* > 0.895.

Absolute values of observer differences ABS (Δ_I_) from *D*_I, MEAN_ ranged from 0.0 to 2.5 mm, the median was 0.8 mm. ABS (Δ_E_) also ranged from 0.0 to 2.5 mm, and the median was 0.7 mm. The relative measurement differences from the mean of the three observations were calculated as: Δ_rel_ = 100·ABS(Δ)/*D*_MEAN_. For *D*_I_, the median of the relative differences Δ_I,rel_ was 1.9%, the maximum 4.7%; for *D*_E_ median Δ_E,rel_ was 2.1%, and the maximum 5.5%.

**Fig. 4:**
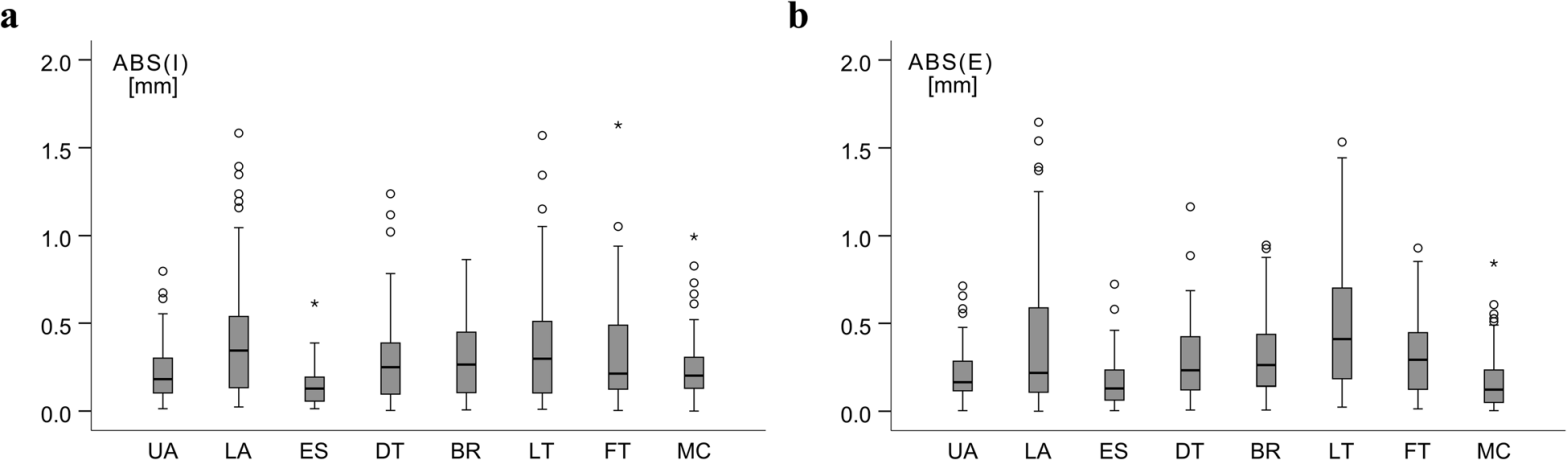
Absolute measurement differences at the individual sites. Absolute differences ABS(δ) of each observer from the mean of the three observers were compared at each site (*n* = 3·20 = 60). **a** ABS (δ_I_) for each of the eight sites including embedded fibrous structures. **b** ABS (δ_E_) for each site excluding embedded fibrous structures. Upper abdomen (UA); lower abdomen (LA); erector spinae (ES); distal triceps (DT); brachioradialis (BR); lateral thigh (LT); front thigh (FT); medial calf (MC). Outliers are shown as circles, extreme values as stars

Figure 4a shows absolute values of observer deviations from their mean ABS (δ_I_) at the eight individual sites (*n* = 3·20 = 60), Fig. 4b shows ABS (δ_E_). Median values of ABS (δ_I_) ranged from 0.1 to 0.3 mm, maximum deviation was 1.6 mm. Median values of ABS (δ_E_) ranged from 0.1 to 0.4 mm, maximum deviation was 1.7 mm [see Additional file 2].

The BMI values ranged from 13.6 to 17.7 kgm^− 2^ (Table 1); Table 3 compares each participant’s *D*_I_ value to the BMI. Pearson’s *r* was 0.78 (*p* < 0.01). Although the BMI range covered only about four units, the highest *D*_I_-value, *D*_I_ = 86.4 mm, was 3.4 times larger than the lowest *D*_I_-value, *D*_I_ = 25.7 mm. In several cases, almost the same BMI values were associated with large differences in *D*_I_, e.g. individuals with a BMI of 15.3 kgm^− 2^, 15.4 kgm^− 2^, and 15. 7kgm^− 2^ showed sums of SAT thicknesses of 32.4 mm, 52.6 mm, and 61.2 mm, respectively.

**Table 3:** Mean SAT thickness sums and anthropometric characteristics for each participant

P	*D*_I,MEAN_ [mm]	BMI [kgm^− 2^]	MI [kgm^− 2^]	*C* [1]
1	25.7	13.6	12.5	57.6
2	32.4	15.3	14.7	55.0
3	32.4	14.7	13.8	56.3
4	36.7	15.2	14.1	56.9
5	37.1	14.7	13.6	57.1
6	38.0	14.2	13.6	55.4
7	39.2	15.4	13.8	59.2
8	39.9	15.7	14.5	57.2
9	40.2	13.8	13.0	56.2
10	43.5	14.5	13.5	56.9
11	44.8	16.7	15.6	56.7
12	48.7	14.2	14.0	53.7
13	52.6	15.4	14.7	55.5
14	52.7	14.7	13.5	57.4
15	52.9	15.6	14.7	56.3
16	53.4	15.6	14.8	55.8
17	61.2	15.7	15.0	55.3
18	64.9	17.7	16.5	56.6
19	78.5	17.5	16.5	56.2
20	86.4	17.7	17.1	55.0

Mean sum of subcutaneous adipose tissue (SAT) including embedded fibrous structures (*D*_I.MEAN_) shown for each of the 20 participants (P), together with the individual body mass index (BMI), mass index (MI), and Cormic index (*C*)

Table 3 also shows the Cormic indices of the children and the improved measure for relative body weight MI, which considers the individual’s leg length. All MI values were lower than the BMI values, indicating shorter leg lengths of children when compared to adults. For randomized groups of Caucasian adults, the mean BMI is equal to the mean MI [4, 12].

## Discussion

This inter-observer study conducted by three observers (of two research centers) in 20 children aged three to six years resulted in a SEE of 1.1 mm, and the 95% limits of agreement were within ±2.1 mm. The ICC was 0.998 (95% CI 0.980–0.999), and the median difference in *D*_I_ was 0.8 mm, i.e. about 1.9% of mean *D*_I_. This is comparable to the high reliability that was previously found in adults [12, 19, 20].

The brightness-mode based ultrasound method for measuring SAT has been standardized [19] and applied to various groups of adults including elite athletes [12, 15, 18, 19, 28], patients with anorexia nervosa [13], and adults with overweight and obesity [20]. Provided that the appropriate speed of sound for adipose tissue is used, accuracy of determining tissue borders is approximately 0.1–0.2 mm at 12–18 MHz probe frequency [15, 19], which cannot be outperformed by any other measurement method due to biological reasons [4].

Previously, this method was used in a pediatric sample for the first time to examine sexual dimorphism of adipose tissue in 274 children aged three to five years. The study found that mean SAT thicknesses significantly differed between boys and girls, even though neither the BMI nor the waist circumference differed [21].

The application of this technique has revealed high intra- and inter-observer reliability in several adult populations [12, 18–20], and high intra-observer reliability in three- to five-year-old children [21]. Table 4 summarizes the results of previous intra- and inter-observer studies comparing the sums of SAT including embedded structures *D*_I_ along with the inter-observer results of the present study. This overview shows that differences in *D*_I_ were about three times as large when measurements were conducted by novices compared to experienced observers [12]. In this previous publication it was found that 95% of experienced observer differences from their mean were less than 1.4 mm.

**Table 4:** Overview of inter-observer and intra-observer reliability studies results

Study reference	Sample	Observers	*D*_I_- range [mm]	95% of values *D*_I_ [mm]	Median of ABS(∆*D*_I_) [mm]
**Inter-observer studies**					
(19)	Adults	EO	10–51	± 1.0	0.2
(12)	Adults	EO	6–70	± 1.2	0.3
(12)	Adults	NO	6–70	± 3.1	1.0
Current	Children	EO	26–86	± 2.1	0.8
**Intra-observer studies**					
(20)	Adults	EO	12–77	± 1.4	0.4
(20)	Adults	EO	44–245	± 2.9	0.9
(20)	Adults	EO	12–245	± 2.2	0.6
(12)	Adults	EO	6–70	± 1.4	0.4
(12)	Adults	NO	6–70	± 3.1	0.6
(21)	Children	EO	35–112	± 2.0	0.9

Studies comparing the sums of subcutaneous adipose tissue (SAT) including embedded structures (*D*_I_). Numbers in parentheses refer to the references. *EO* Experienced observers, *NO* Novice observers, *ABS* Absolute value, ∆*D*_I_ Differences in the sum of SAT thicknesses from the mean of the three of observers

In this sample measured by the three observers, the median absolute value of observer differences in SAT thickness values ABS (δ_I_) was 0.3 mm at each of the sites lower abdomen, lateral thigh, distal triceps, and brachioradialis. However, the medians of the relative values of the differences varied depending on the SAT thickness at the given site: 4.3, 2.9, 3.4, and 6%, respectively. The relative differences were smaller with increasing SAT thickness (Fig. 4; Additional file 2). Similarly, Störchle et al. (2017) found absolute differences at the individual sites to increase with increasing SAT thickness, yet the relative differences decreased with increasing SAT thickness. This was also observed in the sum of SAT thicknesses in the overweight/obese group with larger SAT thickness sums: median Δ_I,rel_ = 0.5%, compared to the leaner group with median Δ_I,rel_ = 1.1% [20]. Obviously, both intra- and inter-observer differences increase with larger SAT thickness sums, however, the relative differences decrease with respect to SAT thickness (Table 4).

Although there was a significant correlation between the BMI and *D*_I_, a comparison of the BMI and *D*_I_ at the individual level revealed substantial differences in SAT thickness sums in several cases, despite a similar BMI (Table 3). For example, two participants who had a difference in BMI of only 0.4 kgm^− 2^, showed a difference in *D*_I_ of about 29 mm: 32.4 mm versus 61.2 mm. This would result in a prediction error of 90% if the BMI was used as a measure of fat. This example (and several more ones in Table 3) points out that the BMI should not be used as a measure of an individual’s body fat [4].

In addition to its high accuracy and reliability, this method has important advantages that are of particular concern when investigating children: minimal subject involvement, no ionizing radiation is applied, fat thickness layers can be quantified across a wide range of thicknesses, many thickness measurements from one image result in small standard errors of the mean thickness at a given site, and it is applicable in the field.

### Limitations

This ultrasound technique measures SAT, but does not include visceral adipose tissue. However, SAT typically amounts to 80–90% of total body fat [29–31] and is therefore a good representative of total body fat.

As this is a new approach to analyse body fat in children, normative values for SAT obtained with this highly accurate and reliable US method do not yet exist, but a comprehensive reference data set can now be collated because this research, together with a previous publication [21], have shown that both intra- and inter-observer reliability are high and comparable to previous findings in adults.

Guided training of observers is necessary to ensure high accuracy and reliability [12, 19]. For a measurer who had some prior ultrasound imaging experience, a two-day course is sufficient to get started. In this study, experienced observers performed the measurements. For the inter-reliability study this research focused on, the number of measurements was large: three observers captured and evaluated 160 images each; a larger number of participants would not have a noticeable effect on the inter-observer reliability results. However, anthropometric and body composition data of this sample of 20 children is not representative for the statistical population. Future studies in various groups of children will be necessary for deriving normative values based on this standardized measurement technique.

Ultrasound is more expensive than other field methods, but is much cheaper and easier to perform in children than other imaging methods such as magnetic resonance imaging or computer tomography.

## Conclusions

The highly accurate brightness-mode US technique for measuring SAT that has been developed for adults can also be applied to young children aged three to six years: no modification of site definitions was necessary in this group. This standardized method measures uncompressed SAT, which accounts for the most of total body fat, on a reliability level comparable to that found in adults previously. Because of the high thickness measurement accuracy (about 0.1 to 0.2 mm), this method is the only one that enables a quantification of fibrous structures (fasciae) embedded in the SAT, which amount to a substantial percentage of the subcutaneous adipose tissue mass. The reliability of SAT thickness measurements when embedded fibrous structures (fasciae) are excluded is comparable to the measurements when these structures are included. This standardized method enables body composition and fat patterning analyses in children on a much finer scale than obtainable with any other method. The reliability results found here indicate that there is high potential for ultrasound to replace or compliment other methods for determining body fat in children. Training is necessary to obtain the high reproducibility and accuracy level possible with this standardized method.

## Supplementary information

**Additional file 1.** SAT thickness sums excluding fibrous structured of each participant measured by the three observers

**Additional file 2.** Absolute differences in SAT thickness values to the mean of the three measurements at each individual site

## Data Availability

All data generated or analyzed during the current study are included in this published article and its’ additional files.

## Abbreviations

WHO: World Health Organisation
BMI: Body mass index
MI: Mass index
SAT: Subcutaneous adipose tissue
*D*_I_: Sum of SAT at eight body sites including embedded fibrous structures
ICC: Intraclass-correlation coefficient
CI: Confidence interval
*d*: Mean SAT thickness at a given site
I: Including embedded fibrous structures
E: Excluding embedded fibrous structures
*D*_E_: Sum of SAT at eight body sites including embedded fibrous structures
SD: Standard deviation
Δ: Difference of the sum of eight sites from the mean of sums measured by three observers
*D*_MEAN_: Mean of sums measured by three observers
ABS: Absolute
Δ_rel_: Relative difference of the sum of eight sites from the mean of sums measured by the three observers
*ϱ*: Empirical mean deviation in measurements from the three observers for each participant at each individual site or each individual sum of SAT
