# Supplementary material for: High inter-observer reliability in standardized ultrasound measurements of subcutaneous adipose tissue in children aged three to six years
Source: BMC Pediatr. 2020 Apr 2;20:145. doi: 10.1186/s12887-020-02044-6 (PMC7114789; doi:10.1186/s12887-020-02044-6)
Supplement: Supplementary file 1 — Additional file 1. SAT thickness sums excluding fibrous structured of each participant measured by the three observers [file 12887_2020_2044_MOESM1_ESM.docx]

Additional file 1- SAT thickness sums excluding fibrous structures for each participant measured by the three observers

|  |  | *D*_E_ [mm] | | | Δ_E_ [mm] | | |
| --- | --- | --- | --- | --- | --- | --- | --- |
| P | ***D*_E, MEAN_** | **OBS1** | **OBS2** | **OBS3** | **OBS1** | **OBS2** | **OBS3** |
| 1 | 21.4 | 21.0 | 21.3 | 21.7 | -0.3 | 0.1 | 0.3 |
| 2 | 28.2 | 28.0 | 28.2 | 28.3 | -0.2 | 0.0 | 0.1 |
| 3 | 28.8 | 28.2 | 28.2 | 29.9 | -0.6 | -0.6 | 1.1 |
| 4 | 32.6 | 32.2 | 32.0 | 33.7 | -0.4 | -0.6 | 1.1 |
| 5 | 33.7 | 33.7 | 33.5 | 34.0 | 0.0 | -0.2 | 0.3 |
| 6 | 34.4 | 33.7 | 34.7 | 34.9 | -0.7 | 0.3 | 0.5 |
| 7 | 34.7 | 33.0 | 35.8 | 35.2 | -1.7 | 1.1 | 0.5 |
| 8 | 35.3 | 35.5 | 35.5 | 34.9 | 0.2 | 0.2 | -0.4 |
| 9 | 36.4 | 35.0 | 37.0 | 37.2 | -1.4 | 0.6 | 0.8 |
| 10 | 38.9 | 37.9 | 40.5 | 38.2 | -1.0 | 1.6 | -0.7 |
| 11 | 38.9 | 39.4 | 37.8 | 39.5 | 0.5 | -1.1 | 0.6 |
| 12 | 43.5 | 42.1 | 43.6 | 44.9 | -1.4 | 0.1 | 1.4 |
| 13 | 46.9 | 45.2 | 49.2 | 46.2 | -1.7 | 2.3 | -0.7 |
| 14 | 47.0 | 45.5 | 47.3 | 48.4 | -1.5 | 0.3 | 1.4 |
| 15 | 48.4 | 47.6 | 47.4 | 50.2 | -0.8 | -1.0 | 1.8 |
| 16 | 48.7 | 49.8 | 48.6 | 47.8 | 1.1 | -0.1 | -0.9 |
| 17 | 56.9 | 54.9 | 58.5 | 57.3 | -2.0 | 1.6 | 0.4 |
| 18 | 60.6 | 60.8 | 60.6 | 60.4 | 0.2 | 0.0 | -0.2 |
| 19 | 73.2 | 73.9 | 74.9 | 70.6 | 0.7 | 1.7 | -2.6 |
| 20 | 80.5 | 79.7 | 81.1 | 80.7 | -0.8 | 0.6 | 0.2 |

Individual thickness sums of subcutaneous adipose tissue (SAT) excluding embedded fibrous structures (*D*_E_) shown for each participant (P) and each observer (OBS), and the means of the three measurements (*D*_E, MEAN_). Individual observer differences from the mean were calculated as: Δ_E_= *D*_E_ - *D*_E,MEAN_.
