# Supplementary material for: High inter-observer reliability in standardized ultrasound measurements of subcutaneous adipose tissue in children aged three to six years
Source: BMC Pediatr. 2020 Apr 2;20:145. doi: 10.1186/s12887-020-02044-6 (PMC7114789; doi:10.1186/s12887-020-02044-6)
Supplement: Supplementary file 2 — Additional file 2. Absolute differences in SAT thickness values to the mean of the three measurements at each individual site [file 12887_2020_2044_MOESM2_ESM.docx]

Additional file 2- Absolute differences in SAT thickness values to the mean of the three measurements at each individual site

| Site | UA_I_ | LA_I_ | ES_I_ | DT_I_ | BR_I_ | FT_I_ | LT_I_ | MC_I_ |
| --- | --- | --- | --- | --- | --- | --- | --- | --- |
| Median [mm] | 0.2 | 0.3 | 0.1 | 0.3 | 0.3 | 0.2 | 0.3 | 0.2 |
| Maximum [mm] | 0.8 | 1.6 | 0.6 | 1.2 | 0.9 | 1.6 | 1.6 | 1.0 |
| Interquartile range [mm] | 0.2 | 0.4 | 0.1 | 0.3 | 0.4 | 0.4 | 0.4 | 0.2 |
| Site | **UA_E_** | **LA_E_** | **ES_E_** | **DT_E_** | **BR_E_** | **FT_E_** | **LT_E_** | **MC_E_** |
| Median [mm] | 0.2 | 0.2 | 0.1 | 0.2 | 0.3 | 0.3 | 0.4 | 0.1 |
| Maximum [mm] | 0.7 | 1.7 | 0.7 | 1.2 | 1.0 | 0.9 | 1.5 | 0.8 |
| Interquartile range [mm] | 0.2 | 0.5 | 0.2 | 0.3 | 0.3 | 0.3 | 0.5 | 0.2 |

Characteristic values of box plots in Figures 4a and b. UA= Upper abdomen; LA= lower abdomen; ES= erector spinae; DT= distal triceps; BR= brachioradialis; LT= lateral thigh; FT= front thigh; MC= medial calf. Index ‘I’ indicates embedded fibrous structures included in the subcutaneous adipose tissue (SAT); index ‘E’ indicates embedded fibrous structures were excluded.
